# Glycemic control and arterial stiffness in a Brazilian rural population: Baependi Heart Study

**DOI:** 10.1186/s13098-015-0082-8

**Published:** 2015-10-06

**Authors:** Rafael de Oliveira Alvim, Carlos Alberto Mourao-Junior, Camila Maciel de Oliveira, Rerisson de Faria Lima, Andréa Roseli Vançan Russo Horimoto, Valéria Aparecida Costa Hong, Luiz Aparecido Bortolotto, José Eduardo Krieger, Alexandre Costa Pereira

**Affiliations:** Laboratory of Genetics and Molecular Cardiology, Heart Institute (InCor), University of São Paulo Medical School, Av. Dr. Enéas de Carvalho Aguiar, 44 Cerqueira César, São Paulo, SP CEP 05403-000 Brazil; Department of Physiology, Federal University of Juiz de Fora, Campus Universitário – Bairro Martelos, Juiz de Fora, Minas Gerais - MG CEP 36036-900 Brazil; Hypertension Unit, Heart Institute (InCor), University of São Paulo Medical School, Av. Dr. Enéas de Carvalho Aguiar, 44, Cerqueira César, Sãos Paulo, SP CEP 05403-000 Brazil

**Keywords:** HbA1c, Arterial stiffness, Glycemic control

## Abstract

**Background:**

Increased arterial stiffness predicts morbidity and mortality, independently of other cardiovascular risk factors, and glycemic control impairments are related to higher vascular stiffness. The aim of this study was to evaluate the association between HbA1c levels and increased arterial stiffness in a Brazilian rural population.

**Methods:**

For this study were selected 1675 individuals (both genders and aged over 18 years) resident in the municipality of Baependi, a city located in the Southeast of Brazil. HbA1c levels were determined by HPLC. Pulse wave velocity (PWV) was measured with a non-invasive automatic device (Complior).

**Results:**

HbA1c levels were associated with an increased PWV. This was more relevant for the third tertile of age. In addition, logistic regression multivariate model including age, blood pressure, gender, BMI and fasting glucose showed that the elevation of a single unit percentage of HbA1c represented an increase of 54 % in the odds of increased arterial stiffness [OR 1.54 (95 % CI 1.01–2.17)]. Both, HbA1c and fasting glucose showed higher discriminatory power in the risk assessment for increased arterial stiffness in the non-diabetic when compared to the diabetic group (AUC of HbA1c = 0.71 vs 0.57, *p* = 0.02; AUC of fasting glucose = 0.66 vs 0.45, p = 0.0007, respectively).

**Conclusion:**

Our findings indicate that a increase in HbA1c levels is associated with increased arterial stiffness and that both, HbA1c and fasting glucose, presented higher discriminatory power in the risk assessment for increased arterial stiffness in the non-diabetic group as compared to diabetic individuals.

## Background

Increased central arterial stiffness is an important determinant of cardiovascular disease (CVD) risk [1]. Epidemiological studies reported that increased arterial stiffness predicts morbidity and mortality, independently of other cardiovascular risk factors [1, 2]. Arterial stiffness measured through carotid-femoral pulse wave velocity (PWV)—a gold standard method—has been associated with measures of subclinical CVD [3]. Moreover, several clinical studies have shown that the arterial stiffness is strongly associated with age, blood pressure [4] and various pathological processes such as hypertension, metabolic syndrome, chronic renal disease, and diabetes [5–8].

Glycated hemoglobin (HbA1c) is a marker of glycemic control that reflects the average blood glucose level over a period of 2–3 months. In addition, the American Diabetes Association published clinical guidelines in which HbA1c level was recommended as a diagnostic test for diabetes [9]. The association between HbA1c levels and coronary heart disease risk has been demonstrated previously [10] and more recently, some studies have shown the association between HbA1c levels and arterial stiffness, measured by PWV, in individuals with and without diabetes [11, 12] and in hemodialysis [13]. However, studies demonstrating the impact of HbA1c levels in the risk of increased arterial stiffness in the general population are sparse.

Based on this scenario and on the clinical applicability of measurements of HbA1c and arterial stiffness, the aim of this study was to evaluate the association between HbA1c levels and increased arterial stiffness in a Brazilian rural population.

## Methods

### Study population

The Baependi Heart Study is a genetic epidemiological study of cardiovascular disease risk factors, with a longitudinal design whose methodology has been previously described [14]. For this study we carried out a cross-sectional analysis of data collected in the second visit of the protocol (between 2010 and 2013). In this study we selected 1675 individuals (both genders and aged 18–102 years) distributed in 109 families resident in the municipality of Baependi, a city located in the Southeast of Brazil. The study protocol was approved by the ethics committee of the Hospital das Clínicas, University of São Paulo, Brazil, and each subject provided informed written consent before participation.

### Anthropometrical Investigations

Anthropometric parameters were measured according to a standard protocol [14]. Height was measured in centimeters and weight in kilograms using a calibrated digital balance. Body mass index (BMI) was calculated as body weight (kg) divided by height squared (m^2^).

### Blood pressure measurements

Blood pressure was measured using a standard digital sphygmomanometer (OMRON, Brazil) on the left arm after 5 min rest, in the sitting position. Systolic (SBP) and diastolic blood pressures (DBP) were calculated from three readings (mean value of all measurements), with a minimal interval of 3 min [14]. Pulse pressure (PP) was calculated as difference between SBP and DBP. The mean blood pressure (MBP) was calculated as DBP plus one-third PP. Hypertension was defined as mean SBP ≥140 mmHg and/or DBP ≥90 mmHg and/or antihypertensive drug use [15].

### Glucose and HbA1c measurements

Fasting glucose were evaluated by standard techniques in 12-h fasting blood samples [16]. HbA1c levels were determined by high-performance liquid chromatography (HPLC) (National Glycohemoglobin Standardization Program, USA). Diabetes mellitus was diagnosed by the presence of fasting glucose ≥126 mg/dL, HbA1c ≥6.5 %, or antidiabetic drug use.

### PWV determination

Carotid–femoral PWV was measured noninvasively by an experienced observer using the *Complior SP*^*®*^ (Artech Medical, Pantin, France). PWV was measurement after each participant had rested for 10–15 min in a supine position. One measurement was made for each participant using as quality control values of tolerance inferior to 5 %. The carotid artery and femoral artery pressure waveforms were recorded by simultaneous assessment of the pulse waves in the right common carotid and femoral arteries as described [17]. The PWV values were obtained based on the direct carotid–femoral distance, the values were then standardized to the ‘real’ carotid–femoral distance by multiplying by 0.8 according to recent recommendations [18]. Because this correction, increased arterial stiffness was defined as PWV ≥10 m/s [18].

### Statistical analysis

Categorical variables were presented as percentage, while continuous variables were presented as mean ± SD. Since there is a well established cutoff for PWV in the literature, we carried out univariate and multivariate logistic regression analysis to determinate the association between HbA1c and increased arterial stiffness, setting PWV >10 m/s as dependent variable, and age, MBP, gender, BMI and glucose fasting as predictor variables. Test for linear trend (*Jonckheere*–*Terpstra test)* was performed by assigning median value for each tertile and treated as continuous variables. The receiver operating characteristic (ROC) curve was built and the area under ROC curve (AUC) was used to measure the discriminatory power in the risk assessment for increased arterial stiffness. Areas under the ROC curves between the markers were compared using a parametric method, with GraphROC for Windows software [19]. *Pearson* correlation coefficients were used to estimate associations between PWV and their confusion variables. Statistical analyses were carried out using SPSS (version 20) software (Chicago, IL, USA), with the level of significance set at 5 %.

## Results

### Demographic data of the study sample

Of the 1675 individuals, 151 (9.0 %) had increased arterial stiffness. The distribution of gender between normal (PWV <10 m/s) and increased (PWV ≥10 m/s) arterial stiffness groups was similar. Hypertension and diabetes frequencies were higher in increased arterial stiffness individuals (Table 1). In addition, demographic and clinical data such as age, BMI, SBP, DBP, MBP, PP, PWV, glucose and HbA1c also were higher in increased arterial stiffness individuals (Table 1).

**Table 1:** Characteristics of subjects stratified by increased arterial stiffness

Characteristics	PWV <10 m/s	PWV ≥10 m/s	*p* value
n	1524	151	*****
Age (years)	42.4 ± 15.3	67.4 ± 10.0	<0.001
Gender, male (%)	40.1	49.7	0.06
Diabetes (%)	5.0	30.5	<0.001
Hypertension (%)	32.9	84.1	<0.001
BMI (kg/m^2^)	25.7 ± 4.8	29.2 ± 29.1	<0.001
SBP (mmHg)	123.7 ± 16.7	142.9 ± 17.2	<0.001
DBP (mmHg)	75.8 ± 10.1	78.3 ± 10.5	0.005
PP (mmHg)	47.9 ± 16.3	64.8 ± 14.6	<0.001
MBP (mmHg)	91.4 ± 10.3	101.7 ± 12.4	<0.001
PWV (m/s)	7.4 ± 1.1	11.7 ± 1.6	<0.001
Fasting glucose (mg/dL)	91.1 ± 17.5	103.5 ± 26.1	<0.001
HbA1c (%)	5.6 ± 0.7	6.3 ± 1.1	<0.001

Hypertension: systolic blood pressure ≥140 mmHg and/or diastolic blood pressure ≥90 mmHg or use of anti-hypertension drugs

Diabetes: fasting glucose ≥126 mg/dL and/or use of hypoglycemic drugs

Increased arterial stiffness: pulse wave velocity (PWV) ≥10 m/s

Continuous data are expressed as mean ± standard deviation

Categorical data are expressed as percentage

*BMI* body mass index, *PWV* pulse wave velocity, *SBP* systolic blood pressure, *DBP* diastolic blood pressure, *MBP* mean blood pressure, *PP* pulse pressure, *HbA1c* glycated hemoglobin

### Correlations between PWV and covariables

*Pearson* correlation coefficients are summarized in Table 2. Age and MBP were the variables that showed the highest correlations with PWV (r = 0.68 and r = 0.46, respectively). In addition, among the variables associated with glycemic control, HbA1c showed a higher correlation with PWV when compared to fasting glucose (r = 0.34 vs r = 0.31, respectively).

**Table 2:** Correlation matrix between age, BMI, fasting glucose, MBP, HbA1c and PWV

Variables	Age	BMI	Fasting glucose	MBP	HbA1c	PWV
Age	1	0.19*	0.34*	0.33*	0.44*	0.68*
BMI	0.19*	1	0.24*	0.23*	0.17*	0.08*
Fasting glucose	0.34*	0.24*	1	0.15*	0.69*	0.31*
MBP	0.33*	0.23*	0.15*	1	0.16*	0.46*
HbA1c	0.44*	0.17*	0.69*	0.16*	1	0.35*
PWV	0.68*	0.08*	0.31*	0.46*	0.35*	1

The level of significance was * p ≤ 0.001

*PWV* pulse wave velocity, *BMI* body mass index, *MBP* mean blood pressure, *HbA1c* glycated hemoglobin

### Test for linear trend and association between HbA1c and increased arterial stiffness

**Fig. 1:**
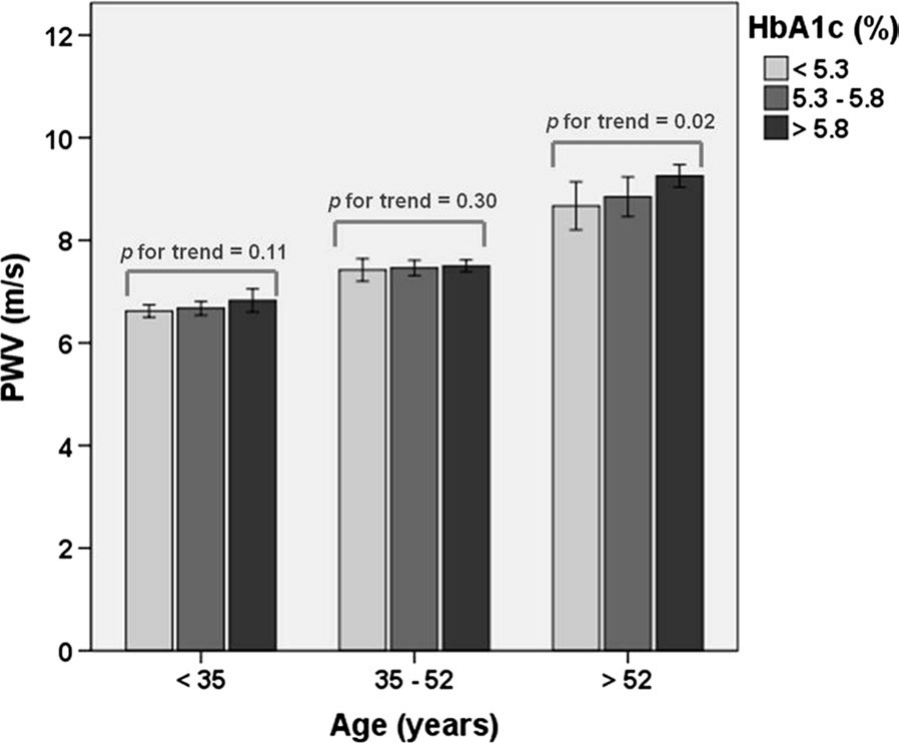
Test for linear trend stratified by age tertile—HbA1c levels were associated with an increasing trend of PWV in a dose-dependent fashion (*p* for trend <0.05)

HbA1c levels were associated with an increasing trend of PWV in a dose-dependent fashion. This was more pronounced in the third tertile of age (*p* for trend = 0.02) (Fig. 1). In this analysis, the difference in mean PWV values between the highest and the lowest tertiles of age was 2.44 m/s.

The univariate logistic regression analysis showed that the elevation of a single unit percentage of HbA1c represented an increase of 119 % in the odds of increased arterial stiffness [OR 2.19 (95 % CI 1.81–2.65)]. In the multivariate model including age, MBP, gender, BMI and fasting glucose, the elevation of a single unit percentage of HbA1c represented an increase of 54 % in the odds of increased arterial stiffness [OR 1.54 (95 % CI 1.01–2.17)] (Table 3).

**Table 3:** Association between HbA1c and increased arterial stiffness by logistic regression univariate and multivariate analysis in a Brazilian rural population

Variable	Increased arterial stiffness	
	OR (95 % CI), *p* value	
	Univariate	Multivariate
HbA1c	2.19 (1.81–2.65), <0.001	1.54 (1.01–2.17), 0.01
Age	1.13 (1.11–1.15), <0.001	1.14 (1.11–1.16), <0.001
MBP	1.08 (1.07–1.10), <0.001	1.07 (1.05–1.09), <0.001
BMI	1.02 (1.01–11.04), 0.04	1.01 (0.99–1.04), 0.28
Fasting glucose	1.02 (1.02–1.03), <0.001	0.99 (0.98–1.01), 0.54
Gender (female)^a^	1.47 (1.05–2.05), 0.03	1.01 (0.63–1.63), 0.95

Increased arterial stiffness: pulse wave velocity (PWV) ≥10 m/s

Multivariate model: HbA1c, age, MBP, BMI, fasting glucose and gender

^a^Female group as reference

### Area under ROC curve of HbA1c and fasting glucose in the diabetic and non-diabetic group

**Fig. 2:**
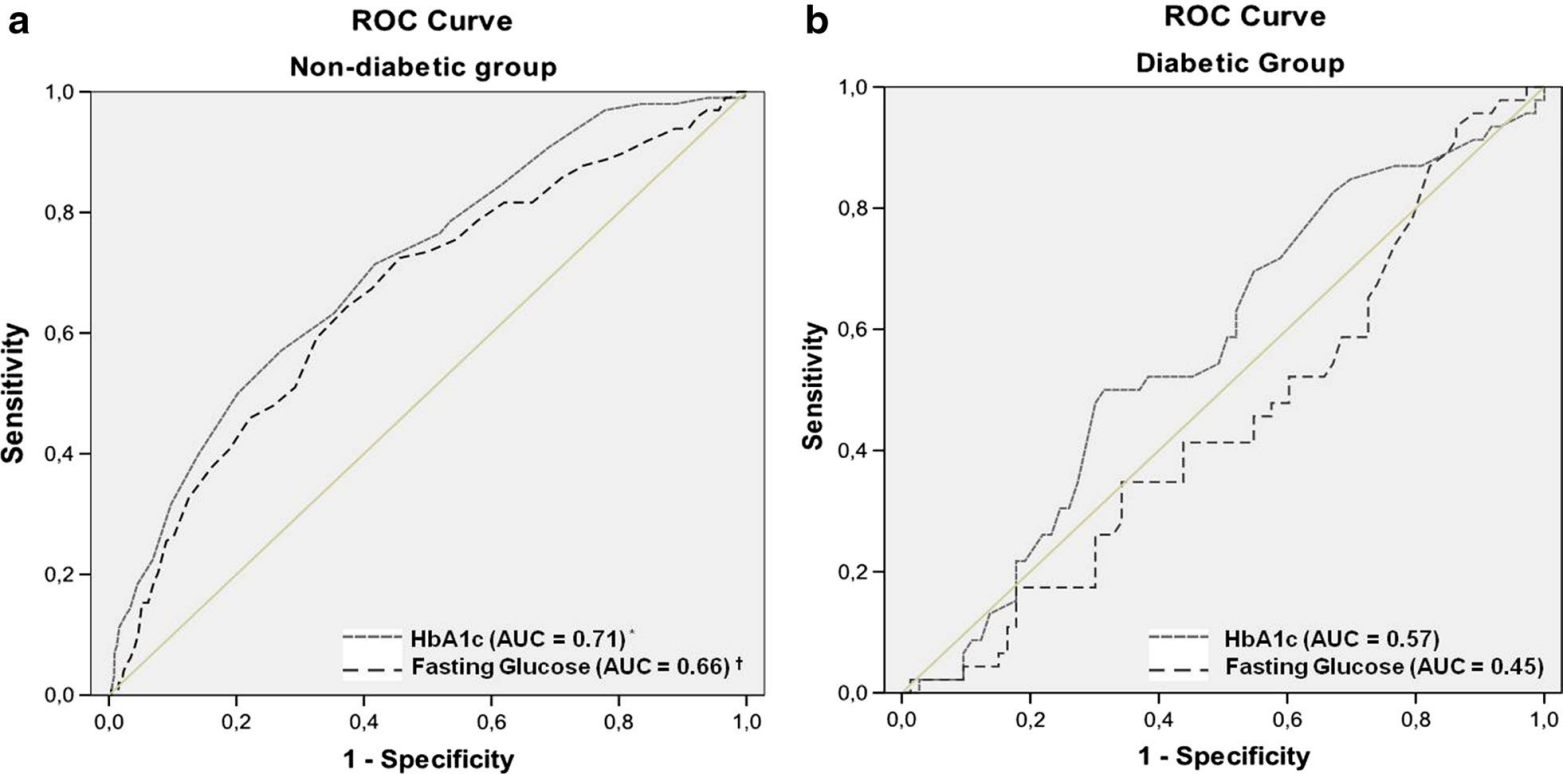
Area under the ROC curve (AUC)—**a** (non-diabetic group) and **b** (diabetic group). Comparison of discriminatory powers of HbA1c and fasting glucose in the risk assessment for increased between diabetic and non-diabetic group. *AUC of HbA1c in the non-diabetic vs the diabetic group (*p* = 0.02). ^†^AUC of fasting glucose in the non- diabetic vs the diabetic group (*p* = 0.0007)

In both, diabetic and non-diabetic groups, the discriminatory powers of HbA1c and fasting glucose in the risk assessment for increased arterial stiffness were not different (AUC = 0.71 vs 0.66, *p* = 0.29; AUC = 0.57 vs 0.45, p = 0.13, respectively). However, the AUC of HbA1c and AUC of fasting glucose were higher in the non-diabetic when compared to the diabetic group (AUC of HbA1c = 0.71 vs 0.57, *p* = 0.02; AUC of fasting glucose = 0.66 vs 0.45, p = 0.0007, respectively) (Fig. 2).

## Discussion

The main finding of our study was the association between HbA1c with arterial stiffness in a Brazilian rural population in which the elevation of a single unit percentage of HbA1c represented an increase of 54 % in the odds of increased arterial stiffness even after adjusting for confounding variables such as age, MBP, BMI, gender and fasting glucose. In addition, the HbA1c presented higher discriminatory power in the risk assessment for increased arterial stiffness in the non-diabetic group.

Several studies have shown that HbA1c predicts cardiovascular risk in people with [20] and without diabetes [12]. Regarding the association of HbA1c with arterial stiffness, Teoh et al. [21], studying 860 individuals from the Edinburgh Type 2 Diabetes Study, showed that PWV was associated with increasing HbA1c, only in the group with mean HbA1c at least 7.6 %. Matsumae et al. [13], studying 242 hemodialysis patients with and without diabetes, showed that HbA1c level was an independent determinant of arterial stiffness, measured by carotid-femoral PWV, in both group. Corroborating such results, Liang et al. [12], studying 5098 Chinese individuals without diabetes, showed that HbA1c levels were significantly associated with an increasing trend of carotid-femoral PWV in a dose-dependent fashion. In our study, HbA1c also was associated to carotid-femoral PWV in which the elevation of a single unit percentage of HbA1c represented an increase 54 % in the odds of increased arterial stiffness even after adjusting for confounding variables such as age, MBP, BMI, gender and fasting glucose. In addition, our study also showed that HbA1c levels were associated with an increasing trend of PWV in a dose-dependent manner in individuals over 52 years (third tertile of age). However, unlike the studies presented above, our sample is composed of individuals from the general population with a more heterogeneous composition of clinical conditions (healthy, diabetic, hypertensive, obese, etc.).

The use of HbA1c as glycemic control and cardiovascular risk marker is widely accepted in diabetic patients [20] and its clinical application in individuals without diabetes has never had great relevance. However, several studies have demonstrated the role of HbA1c in the cardiovascular risk assessment of non-diabetic individuals [10, 12], which extends discussion about the use of HbA1c as a cardiovascular risk marker in individuals from the general population. In our study, HbA1c showed higher nominally discriminatory power in the risk assessment for increased arterial stiffness in the non-diabetic when compared to the diabetic group (AUC of HbA1c in the non-diabetic higher than diabetic group). These data demonstrated that HbA1c can be an important determinant in the risk assessment for increased arterial stiffness in non-diabetics individuals.

The association between excessive glucose exposure, assessed by HbA1c, and arterial stiffness may be explained by several mechanisms such as elevated formation of advanced glycation end products (AGEs) [22], which result in endothelial dysfunction due to the increased generation of reactive oxygen species (ROS), reduced bioavailability of nitric oxide (NO) and induction of inflammation [22–24]. All these mechanisms may contribute to development of cardiovascular disease.

Numerous studies have shown the superiority the HbA1c in relation to fasting glucose as a cardiovascular risk predictor variable [12, 24]. In our study, multivariate logistic regression analysis showed that HbA1c was associated to an increased arterial stiffness odds even after adjustment for fasting glucose in the general population. In addition, despite the discriminatory power in the risk assessment for increased arterial stiffness of either indices (HbA1c and fasting glucose) being similar in both groups (diabetics and non-diabetics), the behavior of glucose is different when dealing with non-diabetic as compared to diabetic individuals (Fig. 2).

Our study has two drawbacks. First, it is a cross-sectional analysis. Therefore, a causal relationship between arterial stiffness and HbA1c could not be established. Second, regarding measurement of HbA1c level, some factors such as kidney failure and uremia could influence the analysis [25]. However, as our study is based in a sample from the general population with low mean age, we think that these factors are not enough to modify our results due to the low prevalence of these two conditions in our cohort.

In summary, our findings indicate that the increase of HbA1c levels are associated with increased arterial stiffness and that both, HbA1c and fasting glucose, presented higher discriminatory power in the risk assessment for increased arterial stiffness in the non-diabetic group. Therefore, we suggest that elevation of HbA1c levels, even within the normal range, could trigger vascular dysfunction in individuals of Brazilian rural population.

## Abbreviations

PWV: pulse wave velocity
AUC: area under ROC curve
CVD: cardiovascular disease
HbA1c: glycated hemoglobin
BMI: body mass index
SBP: systolic blood pressure
DBP: diastolic blood pressure
MBP: mean blood pressure
HPLC: high-performance liquid chromatography
ROC: receiver operating characteristic
AGEs: advanced glycation end products
ROS: reactive oxygen species
NO: nitric oxide
PP: pulse pressure
